# The Electronic Health Record Inbox: Recommendations for Relief

**DOI:** 10.1007/s11606-022-07766-0

**Published:** 2022-08-29

**Authors:** Christine A. Sinsky, Tait D. Shanafelt, Jonathan A. Ripp

**Affiliations:** 1 American Medical Association, Chicago, IL USA; 2 Stanford University, Palo Alto, CA USA; 3 Mount Sinai Health System, New York, NY USA

The inbox has become unbearable. A challenge before the pandemic, the electronic health record (EHR) inbox has become Sisyphean—an involuntary, never-ending, after-hours second job for physicians,^1^ contributing to burnout^2^ and which may lead some physicians to reduce clinical work or leave medicine altogether (a particularly urgent issue given the predicted shortages of physicians which has been exacerbated by the pandemic). In this commentary, we identify the scope and origins of this new workstream, make recommendations to reduce the volume of low-value inbox messages, and suggest practice models and payment modifications to better manage those elements of inbox care that serve the patient.

While some administrative paperwork has long been an unwritten part of the social contract between physicians and regulators, payors and commercial vendors, the advent of the electronic inbox and its direct access to the physician, often unfiltered by staff, has enabled volume to grow dramatically and insidiously. Contributions to the growth in inbox volume arise from multiple sources, including:

- *Federal regulators*: The Department of Health and Human Services’ requirement for the immediate release of test results, without consideration for implications at the point of care, doubled patient portal inquiries regarding potentially sensitive results.^3^
- *Public and commercial payors*: Co-pays for in-person visits (and not for care provided via the patient portal) incentivizes patient use of the portal while requirements for prior authorizations, durable medical equipment renewal signatures, and other gate-keeper functions create work without commensurate compensation.
- *Delivery organizations*: With implementation of EHRs, work previously performed by receptionists, transcriptionists, and others has been transferred to physicians, whose time can appear “free.” Clinical workflows that deploy the physician as the inbox triage agent result in physicians responding to clerical and lower-level clinical inquiries, such as requests for appointment scheduling or dates of last immunization.
- *Commercial pharmacies*: Perhaps less recognized are the contributions of automation by for-profit pharmacy chains, installed to reduce their operating costs, resulting in frequent, redundant auto-requests for prescriptions that have already been renewed.

Pre-pandemic the average family physician spent 1.5 hours per day on the inbox.^1^ At one large integrated healthcare delivery system, family physicians and general internists addressed an average of 100 inbox messages daily during working hours and another 50 each weekday evening.^4^ Analyzing 2019 data for the near complete Epic user base, Holmgren et al. found that US clinicians received nearly 3 times as many inbox messages as clinicians in non-US countries.^5^ Over 1/3 of these inbox messages are system-generated and include many low-value communications, such as automated notifications of tests ordered without results, routine reports of scheduled/canceled/no-show appointments and routine notifications of admission and discharge (without associated clinical information) when a patient has an outpatient procedure such as a colonoscopy.

When the pandemic hit, the model of care delivery was fundamentally altered, as physicians and patients rapidly switched to online care. While only 3% of inbox messages are from patients, such messages often require more physician time per message to safely manage and can be especially time-intensive. The number of patient messages increased by 157% at the onset of the pandemic and have remained at this “new normal” level since.^6^ Now, after two years, we observe that what patients desire and expect is different. Immediate access to one’s physician for non-urgent questions in real time is highly valued and can be valuable; direct access to one’s physician may even be advertised by institutions as a service differentiator in local competitive markets, and yet we have not yet developed the care teams and compensation models designed to deliver care in this way.

The pandemic accelerated a fundamental shift over 2–3 months without the workflows, teamwork, and payment models to make this rise in non-visit-based work manageable. The burden seems to have primarily fallen on physicians. Physicians may now spend hours on clinical e-correspondences which would have been billable time had these conversations occurred in the context of a visit. Meanwhile, the expectations for patient contact hours and RVU productivity have not decreased in response to this growth of between-visit work.

To create the time to address these patient concerns, it is imperative that the inbox be cleared of low value notifications. Healthcare delivery organizations and other stakeholders must design approaches to inbox management that include assessment of the workload and optimized workflows and team responsibilities, along with appropriate resources and staffing to deliver this new dimension of care.

## RECOMMENDATIONS

1. *Measure* the volume of inbox messages: EHR-use metrics can quantify the volume and distribution of inbox work and the time required to manage. Such data can be used to design models of teamwork and compensation aligned with the work. (Researchers, Delivery systems)
2. *Reduce* the volume of inbox messages by turning off low-value notifications, such as that a test was ordered without an available result. Other examples: discontinue routine notification of primary care practices of every test ordered in real time during inpatient care (a concise discharge summary is a better form of communication). Likewise, notify only the ordering physician’s practice of the test result rather than routinely notifying multiple physicians; this also reduces ambiguity about responsibility and the likelihood of missed follow-up. (Delivery systems)
3. Strategically *delegate* remaining messages to a more robust team: Messages that cannot be eliminated by systematic processes should be delegated to an upskilled team member who can research all messages they cannot independently resolve and address these directly with the physician. Optimal team structure (2 clinical support staff per physician in many specialties), stability (same individuals working together each day), and skill level (nurses, or MAs with supplemental training) will facilitate safe and efficient delegation of inbox messages.^7^ (Delivery systems)
4. Provide *payment* for this growing form of medical care and develop evidence-based adjusted expectations for patient contact hours and RVU generation, enabled by optimal staffing ratios. (Payors, Delivery Systems)
5. Advance *research* that quantifies the non-visit-based work across different specialties, the risks and benefits of inbox care, and the effectiveness of interventions meant to reduce inbox burdens. (Researchers, Delivery systems)

The nature of care delivery for patients has fundamentally changed. To a point, diligent physicians took on the expanding inbox work as a contribution to the pandemic mission but that mission is now receding and clinicians may be reaching a breaking point. Design of systems, changes to reimbursement, and staffing are now critical to allow the expansion of care delivery in this way without adding burden to an already overburdened healthcare workforce.

## Declarations

### Disclaimer

Dr. Sinsky is employed by the American Medical Association. The opinions expressed in this article are those of the authors and should not be interpreted as American Medical Association policy.
